# Editorial Perspective: A call for action on imposter participants in child and adolescent mental health research

**DOI:** 10.1111/camh.70041

**Published:** 2025-10-11

**Authors:** Brian C.F. Ching, Cornelius Ani, Angie Pitt, Sharon Eager, Emily Simonoff, Johnny Downs

**Affiliations:** ^1^ CAMHS Digital Lab, Institute of Psychiatry, Psychology & Neuroscience King's College London London UK; ^2^ Department of Child and Adolescent Psychiatry, Institute of Psychiatry, Psychology & Neuroscience King's College London London UK; ^3^ Division of Psychiatry Imperial College London London UK; ^4^ Surrey and Borders Partnership NHS Foundation Trust Leatherhead UK; ^5^ Department of Psychological Medicine, Institute of Psychiatry, Psychology and Neuroscience King's College London London UK; ^6^ Division of Psychiatry University College London London UK; ^7^ NIHR Biomedical Research Centre South London and Maudsley NHS Foundation Trust London UK

**Keywords:** Epidemiology, study design, qualitative methods

## Abstract

Imposter participants are dishonest participants who fake their identities or exaggerate their experiences to participate in research. This appears to be a rising problem in quantitative and qualitative research as well as lived experience groups for child and adolescent mental health research. Imposters in research present great challenges for young people's safety, privacy, and well‐being, and the data integrity of affected research studies. Although strategies have been suggested to identify and address this challenge, they come with limitations. Also, there are no evidence‐based guidelines or frameworks about imposter data. We discuss key issues and considerations and propose actionable steps for researchers and practitioners working with young people. We also make a call for action for robust research to improve how we prevent, identify, and address imposters in child and adolescent mental health research.

**Key Practitioner Message**

- Imposter participants (IPs) present a rising problem such that up to 68% of researchers suspect IPs and fraudulent data in online research
- This issue poses challenges for young people's safety, privacy, and well‐being, and the data integrity of affected research studies
- Current strategies and guidelines to identify and address IPs have limitations and are not evidence‐based
- We highlight key issues for researchers and practitioners to consider during research design, conduct, and dissemination and by reviewers to assess the methodological quality and robustness of the research

## Imposter participants amidst research but unbeknownst?

Imposter participants (IPs) are defined as ‘dishonest participants who completely fake their identities or simply exaggerate their experiences’ to participate in research. The increase in online research methods has provided researchers with the opportunity to advertise their studies and reach wider groups. However, this has also provided imposters with more opportunities to fraudulently and anonymously participate in research.

This phenomenon may be pervasive across any online research opportunity, especially those which involve a payment incentive, including quantitative surveys (Lawlor et al., 2021), qualitative interviews, online focus groups (Pellicano et al., 2024; Pullen Sansfaçon, Gravel, & Gelly, 2024; Ridge et al., 2023), and Patient and Public Involvement and Engagement (PPIE) opportunities. Up to 68% of researchers suspect IPs and fraudulent data in online research (Kumarasamy, Goodfellow, Ferron, & Wright, 2024). Online participation allows imposters the opportunity to search for appropriate answers to interview questions in real time, provide false and nonsensical data in population‐based surveys, access vulnerable groups of young people and their sensitive data in focus groups, or fake different identities to participate multiple times. IPs' motivations to participate fraudulently in research could include financial, sociopolitical, and attention‐seeking.

IPs are especially problematic in mental health research. First, mental health research uses more online qualitative methods (interviews and focus groups) and involves PPIE activities. Second, topics studied and materials covered in research may be particularly sensitive and personal. Finally, those participating may be more vulnerable, including children and young people with mental health problems.

In this editorial, we focus on those who completely feign an identity and/or lived experience to participate in both quantitative and qualitative research. In severe cases, IPs in child and adolescent mental health research may include adults, which could pose direct safeguarding risks to young people involved in group‐based online research.

## Current strategies to identify and mitigate IPs are limited

In recent years, researchers have listed ‘red flags’ to identify potential IPs from personal experiences (e.g., Pellicano et al., 2024; Pullen Sansfaçon et al., 2024; Ridge et al., 2023; Sharma, McPhail, Kularatna, Senanayake, & Abell, 2024), including but not limited to:

- Multiple participant applications within minutes of the research being advertised;
- Identically configured names and email addresses;
- Curt, business‐like communications and multiple follow‐ups;
- Unusually frequent enquiries about incentives;
- Multiple applications from the same IP address, and/or the IP address indicating the participant is not in the country they claim to be;
- Refusal to switch on a video camera;
- Inconsistent demographics (e.g., joining a call with a different name, claiming to be a different age or in a different location);
- Multiple participants with the same voice and identical experiences;
- Irregular background noises;
- Brief, distracted answers;
- Vague or inconsistent accounts of experiences/stories.

However, these potential red flags may not exclusively identify IPs and may also be present among genuine participants. Young people participating in an online interview, focus group, or advisory board may prefer to keep cameras switched off for various reasons, particularly when discussing sensitive topics. Providing brief or inconsistent data and becoming distracted can be common features for some young people (especially neurodivergent youth). Poor internet connection and being motivated by financial incentives may not be uncommon in genuine participants, including those from disadvantaged socioeconomic backgrounds. Thus, only using these potential indicators to identify IPs may inadvertently create further barriers to genuine participation for vulnerable and underrepresented young people in our society.

Additionally, these strategies may only be applicable to qualitative research. Published dialogues around IPs have been predominantly focused on qualitative methodology (e.g., Rees, Liu, Tinker, & Matcham, 2025; Ridge et al., 2023) and less so for quantitative research, where 20%–100% of quantitative survey responses could be fraudulent (Schneider et al., 2024). Many potential signs of IPs may only be identified after interactions with researchers, which may be absent in quantitative approaches. This raises questions about identifying and mitigating imposters in surveys and large‐scale quantitative studies, which are imperative to understanding youth mental health problems and its treatments. Emerging statistical signals that can be used to identify suspicious data, such as clustering or heaping of responses or short survey completion duration, are yet to be evaluated on a large scale.

## Considerations for child and adolescent mental health research

Whilst there is a need to address IPs in all mental health research, there are particular considerations relevant to children and adolescents. In the process of identifying and removing IPs to protect the validity of research, it is important to ensure that young people are not harmed or inadvertently excluded from research. Furthermore, the strategies to address IPs need to retain the core ethical and research principles of respect, beneficence, and inclusion.

Key considerations about IP need to be made by researchers and practitioners during research design, conduct, and dissemination. Similarly, reviewers need to assess what researchers have done to mitigate potential imposter data. We propose considerations to be made about IP in child and adolescent mental health research across sampling and recruitment, systems and processes, data and analysis, and reporting (see checklist at the end of the article). We developed these considerations through discussions with researchers and clinicians who work with young people and consensus‐checked with the King's College London CAMHS Digital Lab. The latter is a multidisciplinary group of researchers, clinicians, and informaticians who integrate operational, clinical, and research expertise to support digital discovery and translate research into practice for child and adolescent mental health. Researchers, practitioners, and reviewers can use the suggested strategies to consider the potential involvement of IPs in their studies pending the development of more comprehensive guidance on this issue. We highlight below issues that can arise when IPs are not properly considered in research.

### Safeguarding, risk, and trust

The infiltration of IPs in online qualitative research may risk the confidentiality and safety of vulnerable minors. Although child and adolescent mental health research will have safeguarding and risk protocols that can be activated whenever harm to participants is suspected, researchers have a responsibility to find strategies to prevent such harm in the first place. Specific safeguarding and risk protocols need to exist to manage suspected IPs in group settings, such as contacting the police (if appropriate) and following up with group members. The unique context of PPIE needs to be considered as they can sometimes be outside usual research ethics and review processes. Moreover, IPs may erode young people and families' trust in researchers and the research process – thereby undermining this key element that underpins research.

### Data minimisation and privacy

Apart from understanding participant characteristics and how these may affect study findings, collecting some personal data helps researchers to confirm a young person's eligibility and to screen out fraudulent participants. However, data minimisation is essential to protect young people's privacy. Thus, robust data collection methods need to have in‐built data minimisation mechanisms to prevent oversharing of information. Also, sensitive information needs to be safely and securely held and destroyed after its purpose for verification is served.

Additional cautions exist for online research in group settings, such as focus groups or PPIE activities. Discussions in these settings may expose sensitive and potentially identifiable details of young people (e.g., full name, the area they live in, the school they attend, medical information). Availability of this personal information and their misuse by IPs could expose the young people to the risk of exploitation, abusive, or predatory behaviour. Thus, protocols specific to group settings need to be carefully developed to protect the privacy and confidentiality of young people's data. For example, young people need to be instructed not to share identifiable information when discussing in groups.

### Inclusion of marginalised groups

Requiring online identity verification or evidence of diagnosis prior to research participation may inadvertently marginalise underserved and vulnerable groups of young people and families. This is also not foolproof for IPs. First, certain groups of young people, such as refugees or asylum seekers, may not have access to relevant identification. Second, disadvantaged families with reduced capacity may already feel disproportionately burdened by research processes, so they may feel discouraged by any extra step such as identity verification. Third, the requirement for evidence of diagnosis is not foolproof as IPs could provide false clinical data. Some of these challenges could be addressed by targeted recruitment through trusted gatekeepers such as charities, mental health services, and schools.

### Concerns about financial incentives

Some IPs may be motivated by financial incentives. However, NIHR INVOLVE guidance states that research should be transparent and value people for their contributions to research. Furthermore, it is important to provide young people and their families with all information about research so they can make informed decisions about whether they want to participate. Sometimes, genuine participants are appropriately incentivised by financial benefits.

### Data integrity

It is challenging when IPs are identified after already interacting with other group members and having provided data in a group setting in qualitative research. Their data can be deleted, but it can be difficult to disentangle their contribution when they may have influenced group discussion. It can be hard to identify IPs in quantitative and large‐scale epidemiological research. Methods to identify fraudulent data may include checking for response times, responses to intentional validity check questions geared towards young people, or the appropriateness of open‐text responses (Schneider et al., 2024). In all cases, researchers need to transparently report evidence of IP in their studies.

## Call for action to develop evidence‐based recommendations and guidelines on IPs in child and adolescent mental health research

IPs present difficult challenges for child and adolescent mental health research. IPs and their fraudulent data could impact the integrity of research findings. In turn, this affects the development of the evidence base for translational research, clinical interventions, and policy. It may also negatively impact practitioners and researchers (Rees et al., 2025) who devote time, resources, and trust to studying important and sensitive topics in young people. Crucially, it also risks young people's safety, privacy, mental health, and trust in research institutions and processes.

Thus, there is an urgent need to develop evidence‐based recommendations and guidelines around IPs. Robust research is needed on the nature of IPs, researchers' experiences of it, and strategies to prevent and address this problem. System‐level support from research organisations such as universities and research ethics boards is required to provide stringent frameworks to prompt consideration of IPs right from the start. There is a need for better support and training for researchers and practitioners who may encounter imposters. Finally, discussions with stakeholders, including young people, are needed about how to prioritise the well‐being of young people whilst preventing IPs and preserving the validity of research.

## Conclusion

IPs are a critical and rising issue for the development of knowledge, clinical care, and the research landscape in child and adolescent mental health. There is an urgent need for strategies to prevent, identify, and address imposters in child and adolescent mental health research. Failure to do so risks undermining the integrity of research and the associated evidence base. IPs also pose a direct risk to young people's safety, privacy, and well‐being. These concerns underline the need for research on IPs and the development of evidence‐based recommendations for addressing this challenge.

## Checklist of domains and specific considerations in relation to imposter participants in child and adolescent mental health research

### Sampling and recruitment

- Discrete sampling for targeted recruitment – includes charities, mental health services, and schools.
- Broader recruitment to widen reach – includes social media, newsletters, community groups, forums, support groups, online networks, and research consortiums.

### Systems and processes

- Method of collecting sensitive and identifiable information that is robust with in‐built data minimisation and secure data protection mechanisms.
- Verification system of young people's identity and/or documents.
- Additional protocol to protect the privacy and confidentiality of young people in online group settings.
- Safeguarding and risk protocol to address suspected IPs in online group settings.

### Data and analysis

- Protocol for data inclusion/exclusion if IP data is suspected or identified.
- Method of checking and cleaning data for data quality control.
- Analysis accounting for potential IP data – includes preliminary analysis and validity checks using statistical or qualitative methods.

### Reporting

- Clear, transparent, and replicable methodology for the prevention and mitigation of potential IPs.
- Characterisation of the signals of suspected IP data.

## Conflict of interest

CA is the Deputy Editor in Chief of Child and Adolescent Mental Health. The other authors have declared that they have no competing or potential conflicts of interest.

## Ethical information

No ethical approval was required for this article.

## Data Availability

Data sharing not applicable to this article as no datasets were generated or analysed.
